# The Impact of Experience with a Family Member with Alzheimer's Disease on Views about the Disease across Five Countries

**DOI:** 10.1155/2012/903645

**Published:** 2012-09-11

**Authors:** Robert J. Blendon, John M. Benson, Elizabeth M. Wikler, Kathleen J. Weldon, Jean Georges, Matthew Baumgart, Beth A. Kallmyer

**Affiliations:** ^1^Department of Health Policy and Management, Harvard School of Public Health, Boston, MA 02115, USA; ^2^Program in Health Policy, Harvard University, Cambridge, MA 02138, USA; ^3^Alzheimer Europe, 145 Route de Thionville, 2611 Luxembourg, Luxembourg; ^4^Government Affairs, Alzheimer's Association, Washington, DC 20005, USA; ^5^Constituent Services, Alzheimer's Association, Chicago, IL 60613, USA

## Abstract

The objective of this paper is to understand how the public's beliefs in five countries may change as more families have direct experience with Alzheimer's disease. The data are derived from a questionnaire survey conducted by telephone (landline and cell) with 2678 randomly selected adults in France, Germany, Poland, Spain, and the United States. The paper analyzes the beliefs and anticipated behavior of those in each country who report having had a family member with Alzheimer's disease versus those who do not. In one or more countries, differences were found between the two groups in their concern about getting Alzheimer's disease, knowledge that the disease is fatal, awareness of certain symptoms, and support for increased public spending. The results suggest that as more people have experience with a family member who has Alzheimer's disease, the public will generally become more concerned about Alzheimer's disease and more likely to recognize that Alzheimer's disease is a fatal disease. The findings suggest that other beliefs may only be affected if there are future major educational campaigns about the disease. The publics in individual countries, with differing cultures and health systems, are likely to respond in different ways as more families have experience with Alzheimer's disease.

## 1. Introduction

Prevalence rates for Alzheimer's disease in Europe and the United States are estimated as some of the highest in the world, growing rapidly with the aging demographic shift [1–4]. Alzheimer's disease is one of the leading causes of death in these countries, and the costs imposed by it are substantial in terms of caregiving demands, lost productivity, and strains on health care systems [5]. Among scientists, health professionals, and policymakers, interest in the disease has grown in recent years due to the growing demands for care by patients and a number of important research findings, including the identification of additional genetic risk factors and the release of new diagnostic guidelines [6, 7]. This complements other recent scientific developments in potential drug therapies, diagnostic procedures [8], and other research on potential risk factors, such as dietary choices, chronic disease, and medication usage [9, 10]. 

Single-country surveys have looked at public attitudes and beliefs about Alzheimer's disease [11, 12]. However, little is known about how levels of awareness and beliefs vary across Europe and the United States, with their different cultures and health systems. To fill this information void, Alzheimer Europe and the Harvard School of Public Health conducted a five-country international poll to assess public understanding about Alzheimer's disease [13, 14]. The poll involved random samples of the public in France, Germany, Poland, Spain, and the US. It is important to look at attitudes and beliefs across countries because results from a single country may not be generalizable to other countries that have different values and experiences with Alzheimer's disease.

The multicountry poll focused primarily on eight broad issues. These included (1) the relative public concern about the disease when compared to other serious national health problems, (2) the public's willingness to see a physician to obtain a diagnosis if they were exhibiting symptoms possibly associated with the disease, (3) public beliefs about whether an effective treatment to slow the progression of the disease was currently available, (4) public beliefs about whether a reliable test was currently available that will determine if a person who is suffering from some confusion and memory loss is in the early stages of Alzheimer's disease, (5) the public's interest in future early diagnostic testing for the disease, should such a test become available, (6) public beliefs about whether Alzheimer's disease is a fatal disease, (7) the level of public awareness of common symptoms that were associated with the disease, and (8) public attitudes about government spending on Alzheimer's research and care. 

The focus of this paper is an analysis of the beliefs and anticipated behavior of those in each country who report that they have had a family member with Alzheimer's disease versus those who say they have not. If the experience of having a family member with Alzheimer's disease affects beliefs and behaviors, overall public attitudes and understanding of the disease might be expected to change as the number of people with experience grows as a result of anticipated demographic shifts. Therefore, understanding the differences in perspective between those who have had a family member with the disease and those who have not is important in predicting possible changes in public beliefs and behaviors in each country. We present the major findings of the five-country poll in this comparative context and suggest implications of the possible changes in public response in the years ahead. 

## 2. Methods

### 2.1. Source of Data

The data are derived from a February 2011 survey by Alzheimer Europe and the Harvard School of Public Health with nationally representative random samples of adults age 18 and older in France, Germany, Poland, Spain, and the United States [13].

### 2.2. Study Design

In each of the five countries, interviews were conducted of both landline telephone numbers using random-digit dialing and cell phone numbers using a list of random cell phone numbers across the country among adults age 18 and older. The average length of interview was 12 minutes. Table 1 shows interview dates, samples sizes, and margins of error at the 95% confidence level for each country. 

The fieldwork was conducted for Alzheimer Europe and the Harvard School of Public Health by TNS, an independent research company based in London, with branches in each of the five countries surveyed. TNS is one of the largest survey research companies internationally and also currently conducts the Eurobarometer surveys of adults in European Union countries for the European Commission.

The interviews were conducted in the language of each country. In the US, interviews were conducted in both English and Spanish.

### 2.3. Poststratification Weighting

Nonresponse in telephone surveys produces some known biases in survey-derived estimates because participation tends to vary for different subgroups of the population. To compensate for these known biases, the sample data are weighted to reflect the actual composition of the adult population in the surveyed countries, calculated on the basis of census data from each country, according to race/ethnicity (US only), age, gender, and region. The sample data are also weighted by telephone status (landline, cell). Other techniques, such as callbacks staggered over times of days and days of weeks and systematic respondent selection within households, are used to help ensure that the sample in each country is representative. 

The data presented in this paper are weighted percentages. After weighting, the sample for each country reflects the demographic composition of the adult population of that country as presented in its census. The results for each country are generalizable to the adult population of that country. 

### 2.4. Statistical Analysis

Data analysis comprises descriptive statistics to ascertain public attitudes on each of the measures. Percentages and confidence intervals (at the 95% confidence level) are shown for the responses to each survey item of those who report that they have had a family member with Alzheimer's disease versus those who say they have not in each country. 

The survey found that the public's reported experience with a family member with Alzheimer's disease varied substantially across the five countries. The proportion ranged from 19% in Poland to 30% in France, 33% in Spain, 34% in Germany, and 42% in the US. 

Table 2 presents comparative data for those with and without experience for the first six issues discussed above.  Table 3 shows comparative data between these two groups on public perceptions of common symptoms associated with Alzheimer's disease.  Table 4 presents comparative data between these two groups on attitudes about government spending related to Alzheimer's disease. We highlight only those differences within each country that are statistically significant (*P* < 0.05).

### 2.5. Limitations

The poll could not identify whether national differences in the proportion of respondents reporting family experience with Alzheimer's disease were related to variation in the incidence of the disease, cultural or historical factors that might influence the use of Alzheimer's disease as a diagnostic term, or differences in public education about the disease in individual countries that might play a role in awareness of the disease. 

Those with family experience with Alzheimer's disease may have received educational support during this experience, which may affect differences in awareness between the experienced and nonexperienced groups.

## 3. Results

### 3.1. Relative Public Concern about Alzheimer's Disease When Compared to Other Serious National Health Problems

Alzheimer's disease is ranked as a major concern in many countries. In three of the countries, Germany, Poland, and the US, those with personal family experience were more likely than those without experience to choose Alzheimer's disease as the disease they were most afraid of getting. Experience did not significantly affect views in France and Spain (Table 2).

### 3.2. Willingness to See a Physician to Obtain a Diagnosis If They Were Exhibiting Symptoms Possibly Associated with the Disease

Most report that they would see a doctor for diagnosis if they had symptoms. There were no differences in any country in those who would seek a physician assessment with signs of possible Alzheimer's disease based on experience with having a family member who had the disease.

### 3.3. Beliefs about Whether an Effective Treatment to Slow the Progression of the Disease Was Currently Available

Many believe an effective treatment to slow the progression of the disease and make symptoms less severe is currently available. In all five countries, experience with a family member did not lead to different beliefs about the current availability of an effective treatment.

### 3.4. Beliefs about Whether a Reliable Test Was Currently Available That Will Determine If a Person Who Is Suffering from Some Confusion and Memory Loss Is in the Early Stages of Alzheimer's Disease

A substantial portion of the public in all five countries believes a reliable test is available to determine if a person suffering from confusion and memory loss is in the early stages of Alzheimer's disease. Only in one country did experience with a family member alter beliefs about the current availability of a reliable test. Belief increased from 44% with no family experience to 56% among those with direct experience in Spain. In all other countries, there was no significant difference between the two groups.

### 3.5. Interest in Future Early Diagnostic Testing for the Disease, Should Such a Test Become Available

There is substantial public interest in early diagnostic testing. There was no difference in any country based on personal experience with a family member in the proportion that said they were very likely to take a diagnostic test without symptoms.

### 3.6. Beliefs about Whether Alzheimer Is a Fatal Disease

Large numbers do not believe Alzheimer is a fatal disease. Experience with a family member increased the belief that Alzheimer was a fatal disease in three countries—France, Poland, and Spain. It did not in Germany and the US.

### 3.7. Awareness of Common Symptoms That Were Associated with Alzheimer's Disease

There is general agreement on some symptoms of Alzheimer's disease, disagreement on others. As shown in Table 3, experience with a family member does not change perceptions of most symptoms across countries. Experience with Alzheimer's disease had no impact on beliefs about three symptoms—hallucinations, problems with pain, and difficulty managing and paying bills.

In three or more countries, experience with a family member affected beliefs about confusion and disorientation, difficulty managing daily tasks, and anger and violence. In each of these cases, experience with a family member increased the belief that these were common symptoms of the disease. In Poland, beliefs about two other symptoms were affected by family experience with the disease. Those who had experience with a family member were more likely than those without experience to believe wandering and getting lost was a common symptom. On the other hand, those who had experience with a family member were less likely than those without experience to believe that difficulty remembering things in their life from years before was a common symptom.

In the US, the belief that difficulty remembering things in their life from the day before was a common symptom was increased by experience with a family member.

### 3.8. Attitudes about Government Spending on Alzheimer's Research and Care

As shown in Table 4, majorities of the public in all five countries favor increased government spending on research on new treatments for Alzheimer's disease and on caring for people who have the disease. In two countries—Germany and Poland—those who had experience with a family member were more likely than those without family experience to favor increased government spending on research on new treatments. Only in Poland, those with family experience were also more likely to favor increased spending on caring for people with Alzheimer's disease.

## 4. Discussion

Obviously, many things could change in the coming years. A single reliable test or a new, effective form of treatment could be found, or certain lifestyle changes could be scientifically recognized as being effective. But in the absence of such developments, one would expect certain changes of attitude and belief to occur as more people have experience with a family member who has Alzheimer's disease. The results suggest that, in general, the public will become more concerned about Alzheimer's disease and more likely to recognize that Alzheimer is a fatal disease, although most beliefs about the disease are unlikely to change.

One of the rationales for this study is that the publics in individual countries, with differing cultures and health systems, are likely to respond in different ways as more families have experience with Alzheimer's disease. What do the survey findings mean for the future in each of the five countries?

For France, we would expect that as the number of people who have experience with a family member who has Alzheimer's disease grows, changes in public attitudes and beliefs would change in three areas. A larger proportion of the French public would believe that Alzheimer is a fatal disease. In addition, more would perceive anger and violence and confusion and disorientation to be common symptoms of the disease.

For Germany, as the proportion of those who have family experience with Alzheimer's disease grows, we would expect changes in three areas. A larger proportion of the German public would choose Alzheimer as the disease they are most afraid of getting. In addition, more would perceive that difficulty managing daily tasks is to be a common symptom of the disease. A larger proportion would also favor increase of government spending on new treatments for the disease.

Poland starts out as the country with the smallest proportion of the population reporting family experience with Alzheimer's disease among the five countries in the survey. As that proportion grows in the future, we would expect changes in public attitudes and beliefs in nine areas. A larger proportion of the Polish public would choose Alzheimer as the disease they are most afraid of getting, believe that Alzheimer is a fatal disease, and perceive the following to be common symptoms of the disease: confusion and disorientation, difficulty managing daily tasks, anger and violence, and wandering and getting lost. If the pattern persists, one would also expect a smaller proportion to perceive difficulty remembering things from their life from years before to be a common symptom. In addition, a larger proportion would be expected to favor increased government spending on research on new treatments for Alzheimer's disease and on caring for people who have the disease. 

For Spain, as the proportion of those who have family experience with Alzheimer's disease grows, we would expect changes in public attitudes and beliefs in three areas. A larger proportion of the Spanish public would believe that a reliable test is available to determine if a person suffering from confusion and memory loss is in the early stages of Alzheimer's disease, believe that Alzheimer is a fatal disease, and perceive anger and confusion to be a common symptom of the disease. 

The United States starts out as the country with the largest proportion of the population reporting family experience with Alzheimer's disease among the five countries in the survey. As that proportion grows in the future, we would expect changes in public attitudes and beliefs in five areas. A larger proportion of the US public would choose Alzheimer as the disease they are most afraid of getting and perceive the following to be common symptoms of the disease: confusion and disorientation, difficulty managing daily tasks, anger and violence, and difficulty remembering things in their life from the day before.

Looking across the five countries, the growing proportion of people who have family experience with Alzheimer's disease is likely to have its greatest impact in Poland. Overall, however, the survey results indicate that most of the public's attitudes and beliefs about Alzheimer in these five countries may not be affected by wider family experience with the disease. These findings show the importance of major educational campaigns about Alzheimer's disease, its diagnosis, treatment, and symptoms. In the absence of such educational efforts, many public attitudes and beliefs are unlikely to change significantly in the future. In addition, without such an educational campaign, some beliefs that are factually incorrect are likely to remain in the public's mind.

These findings are important to each country because they show the possibilities of what widespread educational campaigns could do. Improved public education may contribute to a reduction of personal and social burden in various, for example, helping people plan for the future. The results also suggest that educational campaigns could have a different focus in each country depending on the current level of knowledge, awareness, and beliefs.

With Alzheimer's disease and other forms of dementia becoming a growing global problem [15], these findings suggest that conducting surveys of the public in other countries could provide useful information for health professionals and policy makers dealing with Alzheimer's disease and other forms of dementia in their own country in the future.

## Figures and Tables

**Table 1:** 

	Interview dates	Total interviews	Margin of error (percentage points)
France	February 7–14, 2011	529	±4.3
Germany	February 7–19, 2011	499	±4.4
Poland	February 7–10, 2011	509	±4.3
Spain	February 8–13, 2011	502	±4.4
US	February 7–27, 2011	639	±3.9

**Table 2:** Attitudes and beliefs about Alzheimer's disease, by experience with a family member.

Attitude or belief Country (unweighted *n* for total sample; those who have had a family member with AD; those who have not)	Total % (CI 95%)	Yes, have ever had family member with AD % (CI 95%)	No, have not % (CI 95%)	*P* value (family member versus no family member)
Choose Alzheimer's disease from list of seven diseases as the one they are most afraid of getting				
France (*n* = 529; 166; 363)	27 (23–31)	30 (22–37)	26 (21–31)	0.428
Germany (*n* = 499; 177; 317)	23 (19–27)	31 (23–38)	19 (14–24)	0.015
Poland (*n* = 509; 92; 410)	12 (9–15)	24 (15–33)	9 (6–13)	0.004
Spain (*n* = 502; 172; 328)	24 (20–28)	29 (21–36)	21 (16–26)	0.082
US (*n* = 639; 257; 379)	22 (18–25)	27 (21–33)	18 (14–22)	0.014
If exhibiting confusion and memory loss, you would go to a doctor to determine if cause was Alzheimer's disease				
France (*n* = 529; 166; 363)	88 (85–91)	92 (87–97)	87 (83–91)	0.116
Germany (*n* = 499; 177; 317)	90 (87–93)	89 (84–94)	91 (87–94)	0.637
Poland (*n* = 509; 92; 410)	85 (82–89)	89 (82–96)	85 (80–89)	0.271
Spain (*n* = 502; 172; 328)	95 (93–98)	93 (88–97)	97 (95–99)	0.126
US (*n* = 639; 257; 379)	89 (86–92)	86 (81–91)	91 (88–94)	0.106
Believe an effective treatment currently available to slow the progression of the disease and make symptoms less severe				
France (*n* = 529; 166; 363)	40 (35–44)	45 (37–53)	38 (33–43)	0.183
Germany (*n* = 499; 177; 317)	42 (37–47)	45 (37–53)	40 (34–46)	0.360
Poland (*n* = 509; 92; 410)	63 (58–67)	64 (53–75)	63 (57–68)	0.826
Spain (*n* = 502; 172; 328)	27 (23–31)	29 (22–36)	26 (21–31)	0.510
US (*n* = 639; 257; 379)	46 (42–51)	48 (41–54)	46 (40–51)	0.699
Believe reliable test currently available to determine if a person suffering from confusion and memory loss is in the early stages of Alzheimer's disease				
France (*n* = 529; 166; 363)	50 (46–55)	52 (44–60)	49 (44–55)	0.636
Germany (*n* = 499; 177; 317)	43 (39–48)	48 (39–56)	41 (35–47)	0.216
Poland (*n* = 509; 92; 410)	38 (34–43)	33 (23–44)	40 (34–45)	0.291
Spain (*n* = 502; 172; 328)	48 (43–53)	56 (48–65)	44 (38–50)	0.014
US (*n* = 639; 257; 379)	59 (55–63)	59 (52–65)	60 (54–65)	0.840
Very likely to get diagnostic test if, in the future, one became available that would tell people before they had symptoms whether they will get Alzheimer's disease				
France (*n* = 529; 166; 363)	26 (22–30)	27 (20–34)	26 (21–31)	0.838
Germany (*n* = 499; 177; 317)	23 (19–27)	28 (21–36)	21 (16–26)	0.090
Poland (*n* = 509; 92; 410)	30 (25–34)	35 (25–46)	28 (23–33)	0.227
Spain (*n* = 502; 172; 328)	39 (34–43)	43 (35–52)	36 (30–42)	0.159
US (*n* = 639; 257; 379)	29 (26–33)	31 (25–38)	28 (23–33)	0.420
Believe Alzheimer's is a fatal disease				
France (*n* = 529; 166; 363)	44 (40–49)	53 (44–61)	41 (36–47)	0.023
Germany (*n* = 499; 177; 317)	33 (28–37)	35 (27–43)	31 (26–37)	0.477
Poland (*n* = 509; 92; 410)	34 (30–39)	45 (33–56)	32 (27–37)	0.046
Spain (*n* = 502; 172; 328)	42 (37–46)	51 (43–59)	37 (31–43)	0.007
US (*n* = 639; 257; 379)	61 (57–65)	60 (54–67)	61 (56–67)	0.806

Source: [13].

**Table 3:** Perceptions of common symptoms of Alzheimer's disease, by experience with a family member.

Perceive each of the following to be a common symptom of Alzheimer's disease Country (unweighted *n* for total sample; those who have had a family member with AD; those who have not)	Total % (CI 95%)	Yes, have ever had family member with AD % (CI 95%)	No, have not % (CI 95%)	*P* value (family member versus no family member)
Hallucinations or hearing voices				
France (*n* = 529; 166; 363)	24 (20–28)	23 (16–30)	24 (19–29)	0.910
Germany (*n* = 499; 177; 317)	34 (29–39)	34 (26–42)	34 (28–40)	0.911
Poland (*n* = 509; 92; 410)	26 (22–30)	34 (23–44)	24 (20–29)	0.119
Spain (*n* = 502; 172; 328)	40 (35–45)	44 (36–53)	38 (32–44)	0.185
US (*n* = 639; 257; 379)	40 (36–45)	39 (32–45)	42 (36–47)	0.459
Pain				
France (*n* = 529; 166; 363)	23 (19–27)	20 (13–26)	24 (19–29)	0.283
Germany (*n* = 499; 177; 317)	22 (17–26)	26 (18–34)	19 (14–25)	0.160
Poland (*n* = 509; 92; 410)	21 (17–25)	28 (18–38)	20 (16–24)	0.142
Spain (*n* = 502; 172; 328)	24 (20–28)	20 (14–27)	26 (20–31)	0.196
US (*n* = 639; 257; 379)	25 (21–29)	25 (19–30)	25 (21–30)	0.865
Difficulty managing and paying bills				
France (*n* = 529; 166; 363)	80 (76–83)	83 (77–89)	79 (74–83)	0.297
Germany (*n* = 499; 177; 317)	73 (68–77)	77 (70–85)	71 (65–76)	0.152
Poland (*n* = 509; 92; 410)	73 (69–78)	80 (70–89)	72 (67–77)	0.160
Spain (*n* = 502; 172; 328)	80 (76–84)	82 (76–88)	79 (74–84)	0.434
US (*n* = 639; 257; 379)	84 (80–87)	85 (80–90)	82 (78–87)	0.405
Confusion and disorientation				
France (*n* = 529; 166; 363)	91 (89–94)	95 (92–99)	90 (86–93)	0.027
Germany (*n* = 499; 177; 317)	92 (89–94)	93 (89–97)	91 (87–95)	0.433
Poland (*n* = 509; 92; 410)	86 (83–90)	93 (87–98)	85 (81–89)	0.024
Spain (*n* = 502; 172; 328)	96 (94–98)	97 (95–99)	96 (93–98)	0.403
US (*n* = 639; 257; 379)	92 (90–94)	96 (93–98)	90 (86–93)	0.004
Difficulty managing daily tasks				
France (*n* = 529; 166; 363)	83 (79–86)	86 (80–92)	82 (77–86)	0.247
Germany (*n* = 499; 177; 317)	86 (83–89)	92 (87–96)	83 (79–88)	0.014
Poland (*n* = 509; 92; 410)	85 (81–88)	93 (88–98)	83 (79–87)	0.002
Spain (*n* = 502; 172; 328)	88 (84–91)	91 (86–96)	86 (82–90)	0.171
US (*n* = 639; 257; 379)	88 (85–91)	92 (88–96)	85 (81–89)	0.015
Anger and violence				
France (*n* = 529; 166; 363)	46 (42–51)	55 (47–63)	42 (37–48)	0.013
Germany (*n* = 499; 177; 317)	40 (35–45)	47 (38–55)	37 (31–43)	0.061
Poland (*n* = 509; 92; 410)	35 (31–40)	49 (33–60)	32 (22–37)	0.010
Spain (*n* = 502; 172; 328)	48 (43–53)	56 (48–65)	44 (38–50)	0.023
US (*n* = 639; 257; 379)	53 (49–58)	59 (52–66)	49 (44–55)	0.030
Wandering and getting lost				
France (*n* = 529; 166; 363)	92 (90–95)	95 (92–99)	91 (88–94)	0.072
Germany (*n* = 499; 177; 317)	89 (85–92)	91 (87–96)	87 (83–92)	0.205
Poland (*n* = 509; 92; 410)	88 (84–91)	94 (86–99)	86 (82–90)	0.018
Spain (*n* = 502; 172; 328)	95 (93–97)	97 (94–99)	94 (91–97)	0.197
US (*n* = 639; 257; 379)	91 (89–94)	92 (89–95)	91 (87–94)	0.555
Difficulty remembering things in their life from years before				
France (*n* = 529; 166; 363)	47 (42–51)	44 (36–52)	48 (43–54)	0.405
Germany (*n* = 499; 177; 317)	44 (39–49)	47 (38–55)	43 (37–49)	0.450
Poland (*n* = 509; 92; 410)	58 (54–63)	48 (36–59)	61 (56–66)	0.039
Spain (*n* = 502; 172; 328)	76 (72–80)	70 (63–78)	79 (74–84)	0.056
US (*n* = 639; 257; 379)	68 (64–71)	64 (57–70)	70 (65–75)	0.120
Difficulty remembering things in their life from the day before				
France (*n* = 529; 166; 363)	94 (92–96)	94 (89–98)	94 (92–97)	0.804
Germany (*n* = 499; 177; 317)	87 (84–90)	89 (84–94)	86 (82–90)	0.411
Poland (*n* = 509; 92; 410)	80 (76–84)	85 (78–93)	79 (75–84)	0.179
Spain (*n* = 502; 172; 328)	91 (88–94)	91 (87–96)	91 (87–95)	0.955
US (*n* = 639; 257; 379)	91 (89–94)	95 (92–97)	89 (86–93)	0.020

Source: [13].

**Table 4:** Attitudes about government spending on Alzheimer's research and care, by experience with a family member.

Attitude about spending Country (unweighted *n* for total sample; those who have had a family member with AD; those who have not)	Total % (CI 95%)	Yes, have ever had family member with AD % (CI 95%)	No, have not % (CI 95%)	*P* value (family member versus no family member)
Favor increased government spending on research on new treatments for Alzheimer's disease				
France (*n* = 529; 166; 363)	83 (79–86)	84 (78–91)	82 (77–86)	0.497
Germany (*n* = 499; 177; 317)	68 (63–73)	75 (68–83)	65 (59–71)	0.032
Poland (*n* = 509; 92; 410)	75 (70–79)	85 (76–94)	72 (67–77)	0.013
Spain (*n* = 502; 172; 328)	83 (79–87)	87 (81–93)	82 (77–86)	0.212
US (*n* = 639; 257; 379)	67 (63–71)	66 (60–73)	68 (62–73)	0.758
Favor increased government spending on caring for people with Alzheimer's disease				
France (*n* = 529; 166; 363)	85 (81–88)	83 (77–90)	85 (81–89)	0.599
Germany (*n* = 499; 177; 317)	69 (64–73)	73 (65–81)	67 (61–73)	0.288
Poland (*n* = 509; 92; 410)	71 (67–76)	86 (77–94)	68 (63–73)	0.006
Spain (*n* = 502; 172; 328)	79 (75–83)	82 (76–89)	77 (71–82)	0.187
US (*n* = 639; 257; 379)	60 (56–64)	64 (57–70)	57 (51–63)	0.129

Source: [13].
